# A Path From Childhood Sensory Processing Disorder to Anxiety Disorders: The Mediating Role of Emotion Dysregulation and Adult Sensory Processing Disorder Symptoms

**DOI:** 10.3389/fnint.2019.00022

**Published:** 2019-07-09

**Authors:** Kibby McMahon, Deepika Anand, Marissa Morris-Jones, M. Zachary Rosenthal

**Affiliations:** ^1^Department of Psychology and Neuroscience, Duke University, Durham, NC, United States; ^2^Department of Psychiatry and Behavioral Science, Duke University Medical Center, Durham, NC, United States

**Keywords:** sensory processing, SPD, sensory over-responsivity, sensory under-responsivity, anxiety disorders, emotion regulation, transdiagnostic

## Abstract

Although maladaptive sensory processing has been observed among individuals with persistent heightened anxiety, it is unclear if difficulties processing sensory input early in life lead to anxiety disorders in adulthood and what mechanisms would drive this progression. In a transdiagnostic clinical sample of 231 adults characterized by heightened difficulties with emotion regulation, the present study sought to examine whether: (a) childhood sensory processing disorder (SPD) symptoms predict an increased probability of an anxiety disorder diagnosis in adulthood; and (b) difficulties with emotion regulation and adult SPD symptoms mediate this relationship. Participants were administered the Structured Clinical Interview for Axis-I disorders and self-reported symptoms of SPD experienced in childhood and adulthood. Results suggested that childhood SPD symptoms were significantly associated with a higher likelihood of a lifetime anxiety disorder diagnosis. Difficulties with emotion regulation fully mediated the relationship between childhood SPD and (a) any anxiety disorder in adulthood and, specifically (b) current generalized anxiety disorder (GAD). Further, we found evidence for a candidate model accounting for the relationship among childhood SPD, adulthood SPD, difficulties with emotion regulation, and anxiety disorders in adulthood. Specifically, our data indicated that high symptoms of SPD in childhood may lead to high SPD symptoms in adulthood, which then lead to high emotion dysregulation, ultimately conferring vulnerability for an anxiety disorder diagnosis. Taken together, these findings provide preliminary evidence for how sensory processing impairments in childhood may relate to anxiety through difficulties regulating emotion regulation.

## Introduction

Sensory processing disorder (SPD) is broadly characterized by chronic and significant impairments with the modulation and integration of sensory stimuli. Ayres (1966) was among the first to propose that some individuals suffered from problems with daily functioning due to *sensory integration dysfunction*, or atypical responses to sensory stimuli. More recently, these problems have been recognized as SPD (Miller et al., 2012, 2009) and SPD include patterns of abnormal reactions to sensory input, such as heightened (“over-responsivity”) or reduced (“under-responsivity”) emotional, behavioral or psychological responses to sensory stimuli at normal intensities. Children with SPD often suffer from debilitating social and emotional consequences of their impairments (Ben-Sasson et al., 2009), which may lead to other psychosocial problems. Indeed, symptoms of SPD have been observed among individuals with a wide range of psychiatric problems (Hofmann and Bitran, 2007; Miller et al., 2009; Xiao et al., 2010; Javanbakht et al., 2011; Ahmari et al., 2012; Ferrão et al., 2012; Korostenskaja et al., 2013; Tumkaya et al., 2012; Jaafari et al., 2013). Neither the long-term psychiatric sequelae of childhood sensory processing impairments are known, nor is it known what mechanisms would lead to such problems. As a result, it is unknown whether difficulties processing sensory stimuli early in life lead to a vulnerability in adulthood to mental health problems in general, or to more specific psychiatric disorders. Put differently, the trajectory from childhood SPD symptoms to adulthood is poorly understood. In the absence of research demonstrating the link between SPD and psychiatric symptoms, we also do not know why people with SPD may develop particular psychiatric problems. Because children with SPD will become adults, it is important to gain a better understanding of the relationship between a history of SPD in childhood and (a) associated psychiatric disorders in adulthood and (b) the mechanisms that may confer risk to such disorders. Such work can have an important role in identifying evidence-based treatments and candidate mechanisms of change within treatments to prevent the development of psychopathology stemming from sensory processing dysfunction in childhood.

Previous research has demonstrated a relationship between impaired processing of sensory stimuli and heightened anxiety. Some studies conducted with healthy populations have found associations between self-reported anxiety and over-responsivity to sensory stimuli (Kinnealey and Fuiek, 1999; Kinnealey et al., 2011) as well as under-responsivity (Engel-Yeger and Dunn, 2011). Other studies have extended these findings into clinical populations and found relationships between sensory processing impairments and several specific anxiety disorders, such as generalized anxiety disorder (GAD; Xiao et al., 2010), social anxiety disorder (SAD; Hofmann and Bitran, 2007), obsessive compulsive disorder (OCD; Ahmari et al., 2012; Ferrão et al., 2012; Korostenskaja et al., 2013; Tumkaya et al., 2012; Jaafari et al., 2013), and post-traumatic stress disorder (PTSD; for a review, see Javanbakht et al., 2011). For example, one study found that 41 adults with GAD had deficits in sensory gating, measured by auditory evoked potential P50 amplitudes in response to auditory double clicks (Xiao et al., 2010). Individuals with OCD also have been found to have higher rates of deficits in integrating sensory information (Jaafari et al., 2013), such as the encoding of auditory information (Korostenskaja et al., 2013). Taken together, this research suggests that problems with sensory processing may be related to anxiety transdiagnostically across a range of disorders, and not to any one anxiety disorder specifically. However, it is not clear what processes might account for how sensory processing impairments evolve into anxiety disorders over time.

Symptoms of disordered sensory processing have been linked to emotional functioning in children (Ben-Sasson et al., 2009). For example, one study conducted with a large sample of 1,394 toddler-aged twins found that children with a fearful or anxious temperament were more likely to react with defensiveness towards auditory and tactile stimuli, such as fussing when being groomed (Goldsmith et al., 2006). This relationship has also been found in older children with Asperger’s syndrome, as higher SPD symptoms and anxiety were related within children ages 6–10 and 11–17 years old. Looking at how this relationship unfolds over time, longitudinal studies in infants (Kagan and Snidman, 1991) and toddlers (Pfeiffer et al., 2005) found that high reactivity to sensory stimuli predicts anxious behavior up to 1 year later. These studies suggest that, for some people, there may be a developmental trajectory early in life from sensory processing impairments to problems with childhood anxiety. However, more research is needed to understand if this trajectory also manifests across the lifespan, from childhood to adulthood.

The identification of candidate processes or mechanisms that account for the relationship childhood sensory processing impairments and adult anxiety is necessary to characterize this trajectory and possible intervention targets. Based on studies using longitudinal methods with children (Kagan and Snidman, 1991; Pfeiffer et al., 2005), one potential pathway is that SPD symptoms in childhood may continue into adulthood, over time, contributing to the development of anxiety disorders. In line with this hypothesis, one study with adults with sensory processing impairments tested the effects of a treatment protocol that targeted symptoms related to SPD by increasing awareness of subjective experiences of sensory stimuli and regular exposure to different types of sensory inputs (e.g., rocking chairs, therapy balls) to regulate reactions to aversive stimuli (Pfeiffer and Kinnealey, 2003). Although this was a pilot study with a small sample size (*N* = 15), the authors found that treating reactions to sensory stimuli lead to reductions in anxiety symptoms. This is indirect evidence supporting the possibility that difficulties with sensory processing in adulthood may contribute to the development of problems with anxiety.

Another potential mechanism may be related to the way in which individuals with SPD respond emotionally to aversive sensory cues. Intense, negative emotional reactions to specific sensory stimuli is a central feature of sensory over-responsivity. Indeed, across a range of samples, greater sensitivity to sensory cues has been observed to be related to higher emotional reactivity (McIntosh et al., 1999; Schaaf et al., 2003; Aron and Aron, 1997). As such, the way individuals with SPD regulate their emotional experiences to aversive stimuli may confer higher vulnerability to anxiety disorders over time. *Emotion dysregulation*, or difficulties regulating intense, negative emotional experiences, is central to several forms of psychopathology, including anxiety disorders (for a review, see Hofmann et al., 2012). Problems with emotion dysregulation are a reasonable candidate mechanism to investigate in an effort to better understand the relationship between childhood SPD and anxiety disorders in adulthood.

To begin investigating the long-term course of sensory processing impairments, more research is needed to examine the relationship between childhood SPD symptoms and mental health problems in adulthood. Although longitudinal and epidemiological studies offer highly rigorous methodologies to address this issue, such approaches are expensive, impractical, and need to be informed by additional research. Accordingly, the present study aimed to: (1) evaluate the relationship between symptoms of SPD in childhood and diagnosis of anxiety disorders in adulthood; and (2) examine if SPD symptoms in adulthood and difficulties with emotion regulation account for why some individuals with childhood SPD symptoms do and others do not develop an anxiety disorder in adulthood. For this study, we recruited a transdiagnostic sample of adults to complete self-report and interview measures of SPD symptoms in childhood and adulthood, psychiatric diagnoses, and difficulties with emotion regulation. We hypothesized that endorsing SPD symptoms in childhood would predict a higher likelihood of being diagnosed with an anxiety disorder in adulthood. We also hypothesized that difficulties with emotion regulation and SPD symptoms in adulthood would both mediate the relationship between childhood SPD symptoms and the likelihood of an anxiety disorder diagnosis.

To advance a candidate model for testing in future studies, we explored the sequential nature of the mediating effects of difficulties with emotion regulation and adult SPD. As SPD symptoms in childhood progress to anxiety disorders later in life, it is possible that: (1) children with SPD symptoms develop difficulties regulating their emotions, which may lead to intense, aversive experiences with sensory stimuli in adulthood that are eventually diagnosed as an anxiety disorder; or (2) these children continue to have aversive reactions to sensory stimuli in adulthood, which may lead to more pervasive difficulties regulating emotions that manifest as an anxiety disorder. In the absence of previous literature that provides evidence of either specific trajectory, we evaluated alternative models that test two potential indirect pathways from childhood SPD to anxiety disorders: one with difficulties regulating emotion predicting SPD symptoms in adulthood, and the other with SPD symptoms in adulthood predicting difficulties regulating emotion.

## Materials and Methods

### Participants

Two-hundred and thirty-one participants were recruited in Durham, North Carolina through listservs, newspaper postings, and referrals from mental health providers. Participants also were recruited from a larger study examining difficulties with emotion regulation in adults. Participants were included in the current study if they were: (a) between the ages of 18–65 years; (b) not currently manic; and (c) not currently experiencing an episode of psychosis. Apart from these exclusion criteria, participants were not required to meet any particular diagnostic profile, which allowed us to recruit a transdiagnostic sample.

Participants were primarily female (*n* = 189, 81.8%), Caucasian (*n* = 149, 64.5%), having completed at least some college (*n* = 89, 38.5%) and earning between $10,000 and $65,000 a year (*n* = 99, 42.9%). 54.1% of participants met criteria for an anxiety disorder within their lifetime (*n* = 125). Specifically, participants met criteria for a history of PTSD (*n* = 41, 17.7%), social phobia (*n* = 33, 14.3%), specific phobia (*n* = 10, 4.3%), panic disorder (*n* = 47, 20.3%), agoraphobia (*n* = 6, 2.6%), OCD (*n* = 23, 10.0%), and current GAD (*n* = 77, 33.9%), according to the Structured Clinical Interview for DSM-IV Axis I Disorders (SCID-I; First et al., 1999).

### Measures

#### Structured Clinical Interview for DSM-IV (SCID-I; First et al., 1999)

The SCID-I was used to assess whether participants met criteria for DSM-IV Axis-I disorders. The SCID-I demonstrates high diagnostic accuracy (82%) and inter-rater reliability (0.85 at training and 0.71 at the first quality assurance check; Spitzer et al., 1992; Ventura et al., 1998). To evaluate the inter-rater reliability on psychiatric diagnoses, kappa was calculated using SCID-I diagnoses (*k* = 0.64, *p* < 0.001). Using guidelines from Altman (1999) adapted from Landis and Koch (1977), this finding can be interpreted to indicate a statistically significant moderate strength of agreement between raters. Lifetime anxiety disorder was operationalized by a variable representing the presence or absence of the DSM-IV lifetime diagnosis of panic disorder, PTSD, agoraphobia, social anxiety disorder, specific phobia, OCD, or GAD (according to the SCID-I, GAD was assessed within the past 6 months, while the other disorders were assessed on a lifetime basis).

#### Self-perception of Sensory Reactivity (Rosenthal et al., 2011)

An interviewer-administered measure modeled after validated measures of sensory defensiveness in adults and children (e.g., Adult Sensory Interview; Pfeiffer and Kinnealey, 2003; Short Sensory Profile; McIntosh et al., 1999) was used to obtain reports of reactivity to sensory stimuli in each sensory domain (auditory, gustatory, olfactory, tactile, and visual). Participants were asked to provide examples of bothersome stimuli across sensory domains; after this priming, participants responded to items beginning with the phrase, “Compared to other people” and ending with a sensory example (e.g., are you bothered by car horns) using a Likert type scale (1–10), with higher scores reflecting higher reactivity.

This interview assesses symptoms of SPD in adulthood, and retrospectively in childhood, and adolescence. To assess symptoms of SPD in adulthood, participants respond to interview questions about sensitivity to stimuli across the following domains: auditory (i.e., “are you very sensitive to certain sounds?”), tactile (i.e., “does it bother you to cut/comb/wash/style your own hair?”), gustatory (i.e., “are you very sensitive to the taste, texture, temperature of food?”), olfactory (i.e., “do smells of food or cooking bother you?”), vestibular/proprioceptive (i.e., “do you find certain types of movement unpleasant?”), and visual (i.e., “do bright lights bother you?”). All items assess whether participants experience these sensitivities more intensely or frequently “compared to other people,” in order to capture abnormal sensory processing. Participants respond to these items with a binary response of “yes” or “no.” Responses to all items are combined to yield a total score representing general sensory processing impairments in adulthood (i.e., Adult SPD symptoms). In the present study, Cronbach’s α across all adulthood sensory items was 0.88.

To assess symptoms of SPD in childhood, participants respond to questions about abnormal sensitivity to stimuli in childhood across the following domains: tactile sensitivity (i.e., “When you were a child, how often did you express distress during grooming?”), taste/smell sensitivity (i.e., “avoid certain tastes/smells that are typically part of children’s diets”), under-responsive/seeks sensations (i.e., “enjoy strange noises or seek to make noise for noise sake”), auditory filtering (i.e., “get distracted or have trouble functioning if there was a lot of noise around), visual/auditory sensitivity (i.e., “respond negatively to unexpected or loud noises”), low energy/weak (i.e., “seem to have weak muscles”), and movement (i.e., “become anxious or distressed when feet left the ground”). Participants respond to each item by indicating how frequently they experienced those sensitivities in childhood, on a 5-point Likert-scale that ranged from “always” to “never.” Item scores for each sensory domain in childhood were averaged and combined to yield a total score representing sensory processing impairments in childhood (i.e., Childhood SPD). In the present study, Cronbach’s α across all childhood sensory items was 0.91.

#### Difficulties in Emotion Regulation Scale (DERS; Gratz and Roemer, 2004)

The DERS is a 36-item self-report measure of individuals’ typical levels of emotion dysregulation across six domains: nonacceptance of negative emotions, inability to engage in goal-directed behaviors when experiencing negative emotions, difficulties in controlling impulsive behaviors when experiencing negative emotions, limited access to emotion regulation strategies perceived as effective, lack of emotional awareness, and lack of emotional clarity. Participants respond on a Likert-type scale ranging from 1 (almost never) to 5 (almost always). A psychometric study of the DERS found high internal consistency (Cronbach’s α = 0.93), good test-retest reliability (*r* = 0.88, *p* < 0.01), and adequate construct and predictive validity (Gratz and Roemer, 2004). The total score and subscale scores correspond to sums of relevant items. In the present study, Cronbach’s α for the total score was 0.86.

#### Demographics

A self-report measure was used to obtain demographic and descriptive information, including age, race, income, and years of education.

### Procedures

Participation in this study involved one or two visits to the lab. During the first visit, participants met with a master’s level diagnostic assessor who had been rated to adherence. Upon arrival at the laboratory, participants provided written informed consent in accordance with the Declaration of Helsinki, using protocols approved by the Institutional Review Board of the Duke University Medical Center. Participants then completed diagnostic interviews, including the Structured Clinical Interview for DSM-IV-TR Axis I Disorders (SCID I; First et al., 1999). Participants who only participated in the current study then completed the sensory interview with the assessor in the same visit. Participants who participated in both the current study and the other study on emotion regulation returned to the lab for the second visit to complete the sensory interview. After finishing these interviews, subjects completed self-report questionnaires.

#### Data Analyses

All analyses were conducted using SPSS (version 25). Logistic regression analysis was conducted to investigate whether self-reported symptoms of SPD in childhood predicted an increased probability of having any lifetime DSM-IV diagnosis of an anxiety disorder (including lifetime panic disorder, agoraphobia without panic, specific phobia, social anxiety disorder, PTSD, OCD, or current GAD). Next, provided a significant association between these two variables, two serial, double mediation models were examined using PROCESS, an SPSS macro for path-analysis based modeling (Hayes, 2018). The two models examined are depicted in Supplementary Figures S1, S2, respectively. All possible indirect paths were tested in both models. Additionally, nonparametric bootstrapping was used to test the significance of indirect effects, in which the effect is interpreted as significant if 95% bias-corrected confidence intervals (CIs) for the effect do not include 0 (Preacher and Hayes, 2004, 2008). Mediation analyses were based on 5,000 bootstrapped samples (as recommended by Hayes, 2009) using bias-corrected 95% CIs.

To explore which specific anxiety disorder may be accounting for the relationship between childhood SPD and anxiety, follow up logistic regression analyses were conducted to test the associations between childhood SPD and the diagnosis of each anxiety disorder separately. Finally, the mediation analyses described above were repeated with specific anxiety disorders that were significantly related to childhood SPD.

Potential covariates were examined using logistic regression analyses to identify variables that are significantly related to the diagnosis of an anxiety disorder. Significant variables were included as covariates in all tests that predicted anxiety disorders. In the mediation models, covariates were included in the models predicting both the mediator and the outcome. Missing values were not included in the analyses and the alpha was set* a priori* at a level of 0.05, two-tailed.

## Results

### Participant Characteristics

Demographic characteristics and incidence of lifetime diagnoses of mood and anxiety disorders in the sample are reported in Table 1.

**Table 1:** Participant characteristics.

	Total (*n* = 231)
Age, mean (SD)	31.19 (11.24)
Female, No. (%)	189 (81.8)
Race, No. (%)	
White	149 (64.5)
African American	47 (20.3)
Asian	18 (7.8)
More than one racial group	7 (7.4)
Income Level, No. (%)	
$0−$10,000	97 (42)
$10,001−$65,000	99 (42.9)
>65,001	32 (13.9)
Education Level, No. (%)	
HS graduate or less	16 (6.9)
Vocational or some college	89 (38.5)
College graduate	54 (23.4)
Graduate school (in progress or completed)	72 (31.2)
DSM-IV Mood and Anxiety Disorder Diagnoses, No. (%)	
Major Depressive Disorder	146 (63.20)
Bipolar Disorder	17 (7.36)
Panic Disorder	47 (20.35)
Specific Phobia	10 (4.33)
Obsessive Compulsive Disorder	23 (9.96)
Generalized Anxiety Disorder	77 (33.33)
Social Phobia	11 (4.76)
Post-Traumatic Stress Disorder	41 (17.75)
Alcohol Dependence Disorder	36 (15.6)
Alcohol Abuse Disorder	27 (11.7)
Other Substance Dependence Disorder	44 (19.1)
Other Substance Abuse Disorder	46 (19.9)

*Note: SD, Standard Deviation; HS, High School; DSM-IV, Diagnostic and Statistical Manual-IV. DSM-IV diagnoses represent lifetime diagnoses for all disorders except for GAD, for which the number represents frequency of current diagnoses*.

### Test of Covariates

Potential covariates (i.e., age, sex, race, income, and education level) were examined using logistic regression analyses to determine if they were related to the diagnosis of any lifetime anxiety disorder (Table 2). An odds ratio of 0.36 indicates that male participants were 64% less likely to meet criteria for any lifetime anxiety disorder compared to female participants. Additionally, African American and Asian participants were less likely to meet criteria for an anxiety disorder compared to the reference group (i.e., participants reporting more than one race). Therefore, sex and race were included as covariates in subsequent analyses.

**Table 2:** Effect of covariates on lifetime DSM-IV diagnosis of any anxiety disorder.

Covariate	B	SE	Wald	*P*	OR
Age	−0.015	0.013	1.352	0.245	0.985
Sex (male)	**−1.010**	**0.352**	**8.248**	**0.004**	**0.364**
Race			**21.631**	**<0.001**	
White	−0.718	0.779	0.851	0.356	0.488
African American	**−1.801**	**0.808**	**4.971**	**0.026**	**0.165**
Asian	**−2.708**	**0.904**	**8.980**	**0.003**	**0.067**
Income Level			3.519	0.172	
$0-$10,000	−0.784	0.536	2.143	0.143	0.456
$10,001-$65,000	−0.993	0.531	3.492	0.062	0.370
Education Level			0.208	0.976	
HS graduate or less	−0.167	0.600	0.077	0.781	0.846
Vocational or some college	−0.124	0.350	0.126	0.723	0.883
College graduate	0.000	0.402	0.000	1.000	1.000

*Note: SE, Standard Error; OR, Odds Ratios. Effect of Sex represents category “male” compared with “female,” effects for Race are in relation to “More than one racial group,” effects of Income Level are in relation to income >65,001, effects for Education Level are in relation to “Some graduate training or higher.” Effects significant at p < 0.05 are in bold*.

### Primary Analyses

After accounting for the effect of sex and race as covariates, high childhood SPD symptoms were significantly associated with a greater likelihood of being diagnosed with a lifetime anxiety disorder. Specifically, for every unit increase in SPD symptoms, the odds of being diagnosed with a lifetime anxiety disorder increased by a factor of 1.02 (Table 3).

**Table 3:** Effect of childhood SPD on lifetime diagnosis of any anxiety disorder.

Covariate	B	SE	Wald	*P*	OR

**Step 1**					
Sex	−1.203	0.388	9.589	0.002	0.055
Race			**21.607**	**<0.001**	
White	−0.868	0.803	1.167	0.280	0.420
African American	**−1.991**	**0.836**	**5.681**	**0.017**	**0.137**
Asian	**−2.905**	**0.932**	**9.723**	**0.002**	**0.055**
**Step 2**					
Sex	**−1.335**	**0.402**	**11.008**	**0.001**	**0.263**
Race			**19.500**	**<0.001**	
White	−0.922	0.823	1.254	0.263	0.398
African American	**−2.004**	**0.857**	**5.466**	**0.019**	**0.135**
Asian	**−2.848**	**0.950**	**8.996**	**0.003**	**0.058**
Childhood SPD	**0.020**	**0.009**	**4.924**	**0.026**	**1.020**

*Note: SE, Standard Error; OR, Odds Ratios; SPD, Sensory Processing Disorder. Effect of Sex represents category “male” compared with “female,” effects for Race are in relation to “More than one racial group.” Effects significant at p < 0.05 are in bold*.

**Figure 1:**
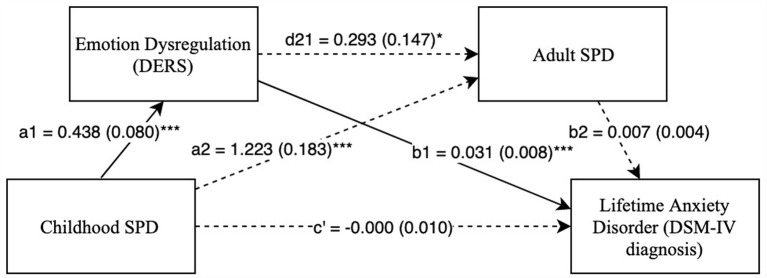
Childhood SPD predicting lifetime anxiety with emotional dysregulation as mediator 1 and adult SPD as mediator 2. Note: *p < 0.05, ***p < 0.001. Standard errors are in parentheses, solid lines represent significant indirect paths, a1 = unstandardized regression coefficient for the IV predicting the mediator 1, a2 = unstandardized coefficient for the IV predicting mediator 2 with mediator 1 in the model, d21 = unstandardized regression coefficient for mediator 1 predicting mediator 2 with IV in the model, b1 = unstandardized regression coefficient for mediator 1 predicting the DV with IV and mediator 1 in the model, b2 = unstandardized regression coefficient for mediator 2 predicting the DV with mediator 1 and the IV in the model, c′ = unstandardized coefficient for the IV predicting the DV with both the mediators in the model (indirect effect). SPD, Sensory Processing Disorder symptoms; DERS, Total score on Difticulty in Emotion Regulation Scale; SPDI, Sensory Processing Disorder Interview, version 2. Sex and race are included as covariates in all regression equations, coefficients of covariates are omitted for clarity.

First, Model 1 (Supplementary Figure S1) was examined with difficulties with emotion regulation as Mediator 1 and adult SPD as Mediator 2. As seen in Figure 1, childhood SPD was significantly associated with higher difficulties with emotion regulation, which in turn significantly predicted a greater incidence of lifetime anxiety disorders when accounting for childhood SPD and adult SPD. Further, when accounting for the two mediators, childhood SPD no longer significantly predicted lifetime anxiety disorders. This pattern of results indicates that difficulties with emotion regulation fully mediated the relationship between childhood SPD and lifetime anxiety disorders. Additionally, the indirect path from childhood SPD → difficulties with emotion regulation → lifetime anxiety disorder was significant (*IE* = 0.014, SE = 0.006, Bias Corrected 95% CI: *LL* = 0.005, *UL* = 0.026)^1^. Because 0 is not included in the CI, these results reveal that the indirect effect of childhood SPD on lifetime anxiety disorders through the mediating effect of difficulties with emotion regulation is significant.

**Figure 2:**
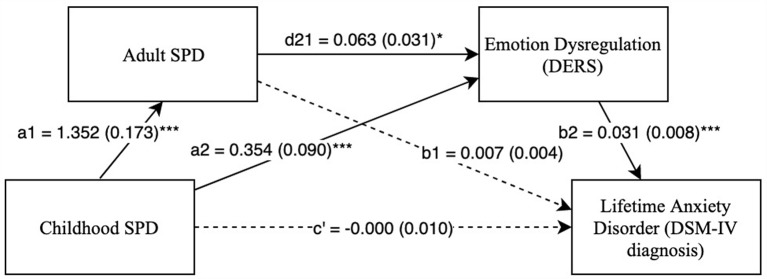
Childhood SPD predicting lifetime anxiety with adult SPD as mediator 1 and emotional dysregulation as mediator 2. Note: *p < 0.05, ***p < 0.001. Standard errors are in parentheses, solid lines represent significant indirect paths, a1 = unstandardized regression coefficient for the IV predicting the mediator 1, a2 = unstandardized coefficient for the IV predicting mediator 2 with mediator 1 in the model, d21 = unstandardized regression coefficient for mediator 1 predicting mediator 2 with IV in the model, b1 = unstandardized regression coefficient for mediator 1 predicting the DV with IV and mediator 1 in the model, b2 = unstandardized regression coefficient for mediator 2 predicting the DV with mediator 1 and the IV in the model, c′ = unstandardized coefficient for the IV predicting the DV with both the mediators in the model (indirect effect). SPD, Sensory Processing Disorder symptoms; DERS, Total score on Difficulty in Emotion Regulation Scale; SPDI, Sensory Processing Disorder Interview, version 2. Sex and race are included as covariates in all regression equations, coefficients of covariates are omitted for clarity.

Next, Model 2 (Supplementary Figure S2) was examined with adult SPD as Mediator 1 and difficulties with emotion regulation as Mediator 2. As seen in Figure 2, the relationship between childhood SPD and lifetime anxiety disorder diagnoses was fully accounted for by two indirect paths: childhood SPD → adult SPD → difficulties with emotion regulation → lifetime anxiety disorders (*IE* = 0.003, SE = 0.002, Bias Corrected 95% CI: *LL* = 0.000^1^, *UL* = 0.007) and childhood SPD → difficulties with emotion regulation → lifetime anxiety disorders (*IE* = 0.011, SE = 0.005, Bias Corrected 95% CI: *LL* = 0.003, *UL* = 0.023). As in Model 1, these results revealed that higher difficulties with emotion regulation fully mediated the relationship between childhood SPD and lifetime anxiety disorders. In addition, there is a significant double mediation such that childhood SPD predicts adult SPD, which then predicts difficulties with emotion regulation, which in turn predicts lifetime anxiety disorders.

Follow up analyses were conducted to test the associations between childhood SPD and each anxiety disorder separately (Table 4). These analyses revealed a significant association with GAD, such that after accounting for race and sex, every unit increase in childhood SPD was associated with an increased likelihood of GAD by a factor of 1.02. Additionally, a unit increase in SPD was associated with a reduced likelihood (by 0.05%) of meeting criteria for Specific Phobia. No significant associations were observed for the remaining anxiety disorders.

**Table 4:** Effect of childhood SPD on lifetime diagnosis of specific DSM-IV anxiety disorders.

Covariate	B	SE	Wald	*P*	OR
Panic Disorder	−0.010	0.009	1.156	0.282	0.990
Specific Phobia	**−0.046**	**0.023**	**4.101**	**0.043**	**0.955**
Obsessive Compulsive Disorder	0.012	0.011	1.107	0.293	1.012
Generalized Anxiety Disorder	**0.018**	**0.008**	**5.275**	**0.022**	**1.018**
Social Phobia	0.015	0.010	2.252	0.133	1.015
Post-traumatic Stress Disorder	0.013	0.009	2.011	0.156	1.013

*Note: SE, Standard Error; OR, Odds Ratios; SPD, Sensory Processing Disorder. Table represents effects estimated in Step 2 of the logistic regression model after accounting for the effect of race and sex as covariates in Step 1. Effects significant at p < 0.05 are in bold*.

**Figure 3:**
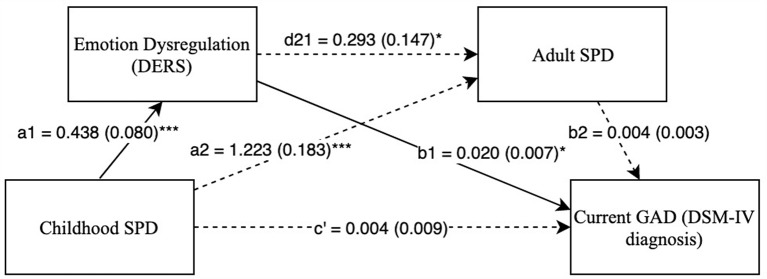
Childhood SPD predicting current GAD with emotional dysregulation as mediator 1 and adult SPD as mediator 2. Note: *p < 0.05, ***p < 0.001. Standard errors are in parentheses, solid lines represent significant indirect paths, a1 = unstandardized regression coefficient for the IV predicting the mediator 1, a2 = unstandardized coefficient for the IV predicting mediator 2 with mediator 1 in the model, d21 = unstandardized regression coefficient for mediator 1 predicting mediator 2 with IV in the model, b1 = unstandardized regression coefficient for mediator 1 predicting the DV with IV and mediator 1 in the model, b2 = unstandardized regression coefficient for mediator 2 predicting the DV with mediator 1 and the IV in the model, c′ = unstandardized coefficient for the IV predicting the DV with both the mediators in the model (indirect effect). SPD, Sensory Processing Disorder symptoms; DERS, Total score on Difficulty in Emotion Regulation Scale; SPDI, Sensory Processing Disorder Interview, version 2. Sex and race are included as covariates in all regression equations, coefficients of covariates are omitted for clarity.

**Figure 4:**
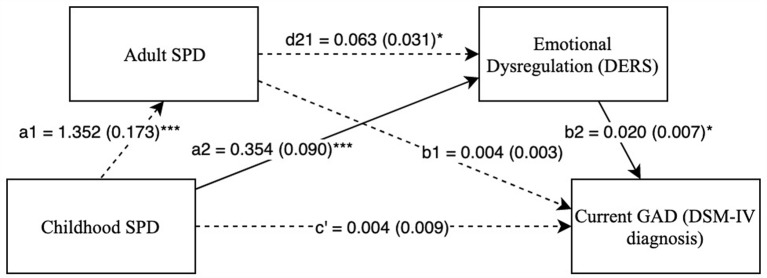
Childhood SPD predicting current GAD with adult SPD as mediator 1 and emotional dysregulation as mediator 2. Note: *p < 0.05, ***p < 0.001. Standard errors are in parentheses, solid lines represent significant indirect paths, a1 = unstandardized regression coefficient for the IV predicting the mediator 1, a2 = unstandardized coefficient for the IV predicting mediator 2 with mediator 1 in the model, d21 = unstandardized regression coefficient for mediator 1 predicting mediator 2 with IV in the model, b1 = unstandardized regression coefficient for mediator 1 predicting the DV with IV and mediator 1 in the model, b2 = unstandardized regression coefficient for mediator 2 predicting the DV with mediator 1 and the IV in the model, c′ = unstandardized coefficient for the IV predicting the DV with both the mediators in the model (indirect effect). SPD, Sensory Processing Disorder symptoms; DERS, Total score on Difficulty in Emotion Regulation Scale; SPDI, Sensory Processing Disorder Interview, version 2. Sex and race are included as covariates in all regression equations, coefficients of covariates are omitted for clarity.

The two serial, double mediation models (Model 1 and Model 2) were examined with GAD as a DV. As seen in Figures 3, 4, there were significant indirect paths between childhood SPD and GAD through difficulties with emotion regulation in both Model 1 (*IE* = 0.009, SE = 0.004, Bias Corrected 95% CI: *LL* = 0.003, *UL* = 0.017) and Model 2 (*IE* = 0.007, SE = 0.003, Bias Corrected 95% CI: *LL* = 0.002, *UL* = 0.015). This suggests that high childhood SPD symptoms account for greater difficulties with emotion regulation, which in turn predicts a higher probability of a GAD diagnosis. Furthermore, after accounting for both mediators, the relationship between childhood SPD and GAD was no longer significant, indicating full mediation through difficulties with emotion regulation. However, indirect paths with SPD in adulthood as a mediator were not significant. Supplementary Figures S3, S4 show the mediation models predicting Specific Phobia as a DV. No indirect paths were significant in these models.

## Discussion

The present study recruited a transdiagnostic sample of adults to cross-sectionally investigate the relationship between self-reported SPD symptoms in childhood and the likelihood of meeting full diagnostic criteria for an anxiety disorder in adulthood. This study also examined difficulties with emotion regulation and adult symptoms of SPD as candidate mediators accounting for the relationship between childhood SPD symptoms and a diagnosis of an adult anxiety disorder. Consistent with our hypotheses, high childhood SPD symptom severity was significantly associated with a greater likelihood of being diagnosed with a lifetime anxiety disorder. Further, these results revealed that difficulties with emotion regulation fully mediated the relationship between childhood SPD and lifetime anxiety disorders. In addition, there was a significant, serial, double mediation in which difficulties with emotion regulation and adult SPD symptoms fully mediated the relationship between childhood SPD and lifetime anxiety disorders. Results from follow-up analyses with specific anxiety disorders revealed that difficulties with emotion regulation also fully mediated the relationship between childhood SPD symptoms and GAD. These findings lend preliminary support to a possible developmental model wherein problems regulating emotions and adult SPD symptoms are target mechanisms through which children with SPD may develop anxiety later in life.

We found that symptoms of self-reported SPD symptoms in childhood significantly predicted the likelihood of meeting full criteria for an anxiety disorder on a lifetime basis. This relationship was also found between childhood SPD symptoms and meeting criteria for GAD within the 6 months prior to the study. These findings are in line with previous research demonstrating that anxiety is related to sensory processing impairments across a range of age groups, from childhood (Kagan and Snidman, 1991; Pfeiffer et al., 2005; Goldsmith et al., 2006; Ben-Sasson et al., 2009) to adulthood (Hofmann and Bitran, 2007; Kinnealey and Fuiek, 1999; Xiao et al., 2010; Engel-Yeger and Dunn, 2011; Kinnealey et al., 2011). However, the present study extends previous research by demonstrating that higher symptoms of SPD in childhood are related to clinically significant levels of anxiety in adulthood in a psychiatrically transdiagnostic sample.

Findings from both mediation models suggested that difficulties with emotion regulation and SPD symptoms in adulthood fully accounted for the relationship between childhood SPD symptoms and meeting criteria for a lifetime anxiety disorder. Further, we found that there was a significant indirect pathway through both mediators, in which high symptoms of SPD in childhood lead to high SPD symptoms in adulthood, which then leads to problems regulating emotions, ultimately leading to a higher likelihood of an anxiety disorder diagnosis. Only difficulties with emotion regulation accounted for the relationship between childhood SPD and a diagnosis of GAD. Findings from the present study support the hypothesis that sensory processing impairments in childhood may be associated with future problems with anxiety through difficulties managing emotional distress. These findings are consistent with Hofmann et al.’s (2012) model of emotion dysregulation and anxiety disorders. This model proposes that individuals who experience aversive events that trigger negative emotions may attempt to regulate those emotions with maladaptive strategies. Over time, this pattern leads to frequent and intense states of dysregulated negative emotions that interfere with daily functioning, which, over time, develop into an anxiety disorder (Hofmann et al., 2012). This model proposes that individuals with a diathesis of higher sensitivity (e.g., SPD symptoms) are more likely to experience these patterns, putting them at higher risk for anxiety disorders. This hypothesis is consistent with findings from studies investigating the neurophysiology of SPD suggesting that people with sensory processing impairments may have heightened autonomic arousal (McIntosh et al., 1999; Schaaf et al., 2003) and amygdala activation (Kagan, 2001) in response to specific sensory stimuli, placing them at risk for the development of anxiety disorders.

Previous research has shown that people with sensory processing impairments may respond to aversive sensory stimuli with maladaptive ways of coping, such as avoidance or withdrawal (Lane et al., 2000). Greater sensitivity to sensory cues is related to higher probability of avoidance of aversive stimuli in the environment (Hofmann and Bitran, 2007), which is in turn related to increased likelihood of state anxiety (Engel-Yeger and Dunn, 2011) or symptoms of specific anxiety disorders such as social anxiety (Hofmann and Bitran, 2007). As such, and in line with Hofmann et al.’s (2012) model, difficulties with emotion regulation may be a key mechanism through which, over time, responses to aversive sensory stimuli may lead to the development and maintenance of anxiety disorders.

Findings from our double mediation model provide evidence of a possible developmental trajectory in which children who have aversive reactions to sensory stimuli are likely to continue to have these experiences later in life. How they cope with those negative experiences may generalize to broader difficulties with regulating emotional reactions to a wide range of stimuli, which may subsequently manifest as clinically significant levels of anxiety. These findings have notable clinical implications, as improving emotion regulation skills and coping with aversive sensory stimuli may prevent children with SPD from developing pathological levels of anxiety. Because difficulty with emotion regulation is a complex construct, future research efforts may identify which specific problems regulating emotions are most related to SPD, therein leading to interventions targeting these problems with specific emotion regulation skills designed to help prevent long-term mental health consequences.

In the present study, childhood SPD symptoms only increased the likelihood of meeting criteria for GAD and did not significantly increase the likelihood of other types of disorders that have been found to relate to sensory processing impairments, such as OCD (Ahmari et al., 2012; Ferrão et al., 2012; Korostenskaja et al., 2013; Tumkaya et al., 2012; Jaafari et al., 2013), and PSTD (Javanbakht et al., 2011). Additionally, childhood SPD symptoms were associated with lower likelihood of meeting criteria for specific phobia. One potential explanation for these findings is that this preliminary study was not powered to detect associations with specific anxiety disorders. GAD was the most prevalent anxiety disorder in our sample (33.9%); other anxiety disorders may have had too low of a prevalence to examine relationships with childhood SPD. Future studies with adequately powered samples are needed to more rigorously investigate the relationship between childhood SPD symptoms and other specific psychiatric disorders.

This study has several key limitations that must be considered when interpreting the results. A primary limitation is the cross-sectional design with retrospective self-reports of SPD symptoms in childhood using an interview measure with limited established psychometric properties. The potential for self-report bias may have applied especially to the measure of childhood SPD symptoms. Additionally, the majority of the items this measure assess for sensory over-responsivity with only a few items assessing other types of SPD patterns, such as sensory seeking or under-responsivity. Therefore, our findings may be driven by sensory over-responsivity symptoms and do not distinguish the relationships with the hypothesized subtypes of SPD that have been proposed in the literature (Miller et al., 2012) or with sensory processing styles that are context-specific (Gepner and Mestre, 2002). Further, the cross-sectional design also does not allow us to determine the temporal nature of these associations. Longitudinal or experimental approaches are needed to determine whether these relationships are causal. For example, future experimental studies can test whether treating sensory processing impairments in adulthood leads to subsequent reductions in emotion dysregulation. Researchers may also investigate whether treatment of difficulties with emotion regulation in adulthood moderates adult SPD. Further, prospective studies are needed to more definitively characterize the developmental trajectory of childhood SPD into adulthood using more dynamic and objective biological measures of sensory processing (e.g., Torres et al., 2013). A second critical limitation is the overrepresentation of females (81.8%) and individuals diagnosed with GAD. Given this sampling bias, these results may not generalize to populations that are predominantly male or suffer from other types of disorders. Third, we assessed SPD symptoms with one interview measure that has only been used in one previous study and therefore has limited psychometric validation. Given these limitations, our findings must be replicated in studies that measure SPD symptoms, anxiety and emotion dysregulation over time with larger samples and validated, multi-method assessments using a combination of interview and objective neurobehavioral or physiological measures.

Despite the study limitations, this is the first study to identify candidate mediators accounting for the relationship between SPD symptoms in childhood and anxiety disorders in adulthood. We found that sensory processing impairments in childhood may increase the risk of anxiety disorders through difficulties with emotion regulation or SPD symptoms in adulthood. These findings provide preliminary evidence of the long-term psychological consequence of childhood SPD symptoms, helping to provide a clearer picture of what psychiatric sequelae may be expected during adulthood in those with childhood SPD symptoms. If our findings are replicated using larger samples and prospective multi-method designs, interventions could be developed to prevent the onset of adult anxiety disorders by targeting difficulties with emotion regulation.

## Ethics Statement

Participants provided written informed consent in accordance with the Declaration of Helsinki, using protocols approved by the Institutional Review Board of the Duke University Medical Center.

## Author Contributions

All authors contributed to the intellectual development and writing of this manuscript. MR, KM and DA conceptualized, wrote, revised and synthesized revisions of this manuscript. MM-J revised sections of the manuscript.

## Conflict of Interest Statement

The authors declare that the research was conducted in the absence of any commercial or financial relationships that could be construed as a potential conflict of interest.

## Footnotes

^1^Three decimal points are included in-text for consistency. The lower limit of CI was 0.0001, indicating significance.
