# Supplementary figures and images for: A Path From Childhood Sensory Processing Disorder to Anxiety Disorders: The Mediating Role of Emotion Dysregulation and Adult Sensory Processing Disorder Symptoms
Source: Front Integr Neurosci. 2019 Jul 9;13:22. doi: 10.3389/fnint.2019.00022 (PMC6629761; doi:10.3389/fnint.2019.00022)

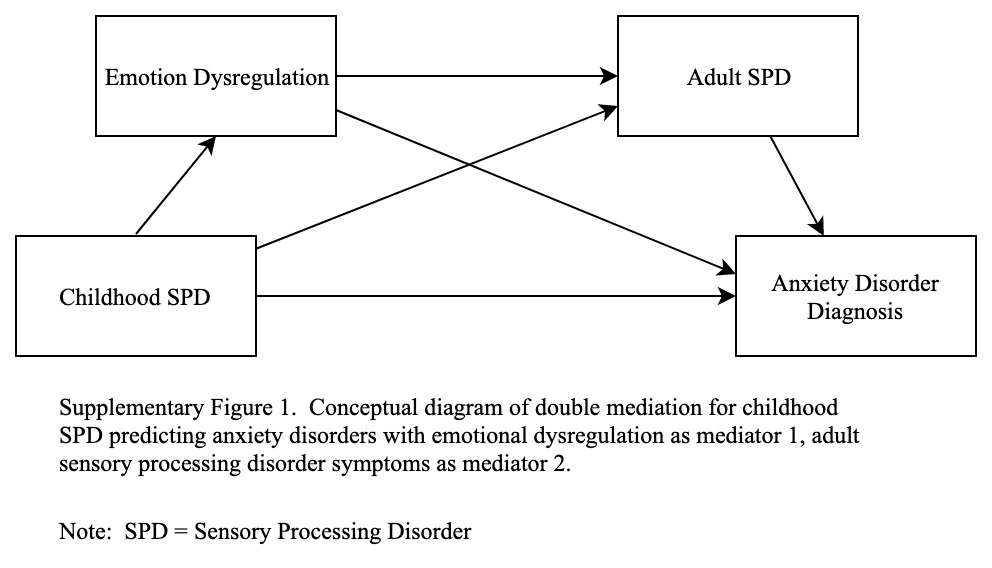

Supplement: Supplementary file 1 [file Image_1.jpg]

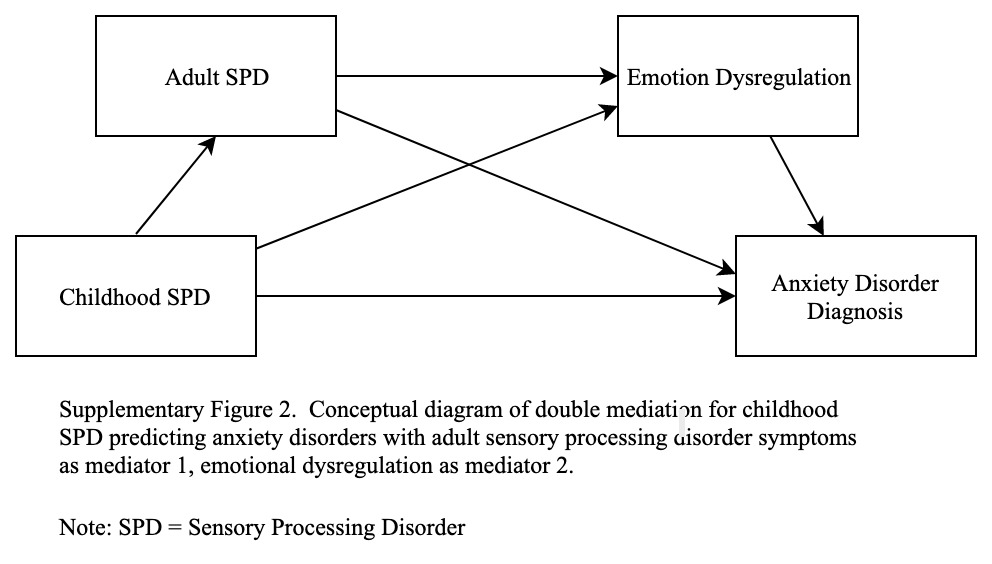

Supplement: Supplementary file 2 [file Image_2.jpg]

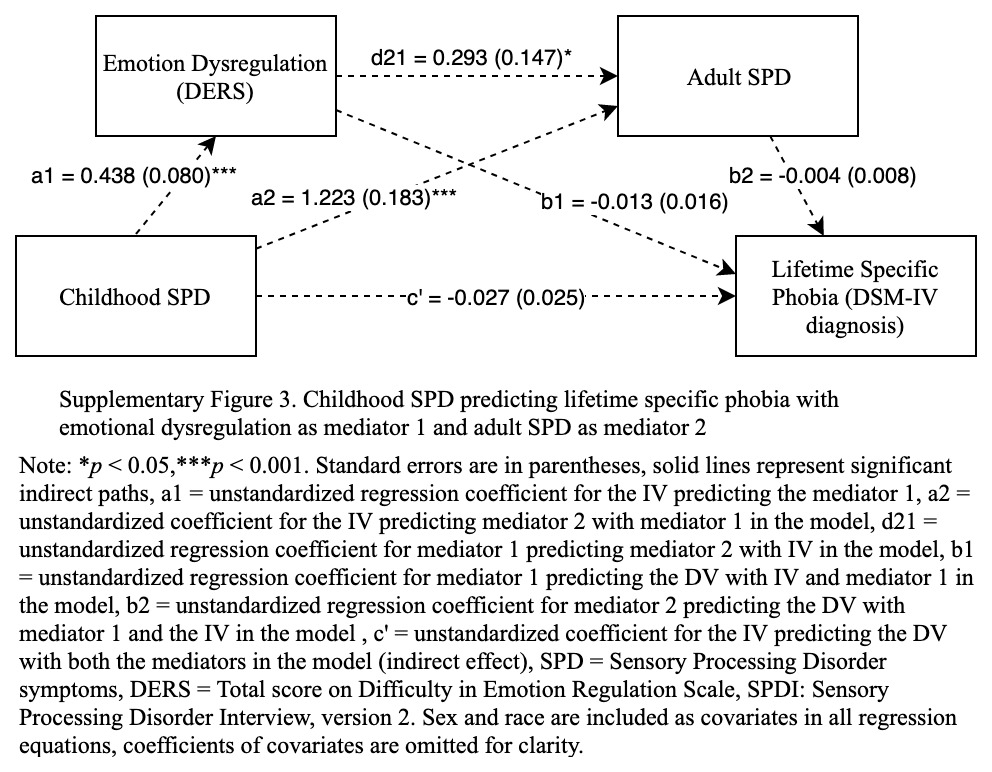

Supplement: Supplementary file 3 [file Image_3.jpg]

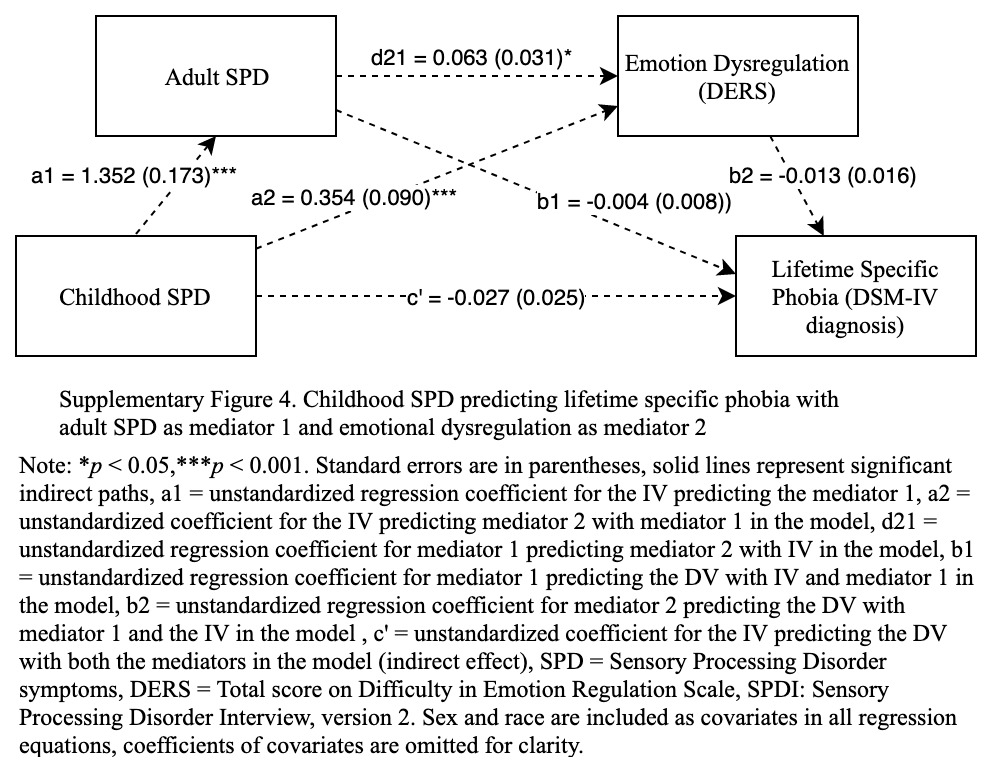

Supplement: Supplementary file 4 [file Image_4.jpg]
